# Correlation of respiratory symptoms and spirometric lung patterns in a rural community setting, Sindh, Pakistan: a cross sectional survey

**DOI:** 10.1186/1471-2466-12-81

**Published:** 2012-12-18

**Authors:** Imran Naeem Abbasi, Adeel Ahsan, Asaad Ahmed Nafees

**Affiliations:** 1 Community Medicine residents, Department of Community Health Sciences, The Aga Khan University, Karachi, Pakistan; 2 Division of Environmental Health Sciences, Department of Community Health Sciences, The Aga Khan University, Stadium Road, P.O.Box-3500, Karachi, Pakistan

**Keywords:** ATS questionnaire validity, Respiratory symptoms, Impaired lung function, Sindhi, Rural community

## Abstract

**Background:**

Symptom-based questionnaires can be a cost effective tool enabling identification and diagnosis of patients with respiratory illnesses in resource limited setting. This study aimed to determine the correlation of respiratory symptoms and spirometric lung patterns and validity of ATS respiratory questionnaire in a rural community setting.

**Methods:**

This cross sectional survey was conducted between January – March 2009 on a sample of 200 adults selected from two villages of district Khairpur, Sindh, Pakistan. A modified version of the American thoracic society division of lung disease questionnaire was used to record the presence of respiratory symptoms. Predicted lung volumes i.e. forced vital capacity (FVC), forced expiratory volume in one second (FEV1) and their ratio (FEV_1_/FVC) were recorded using portable spirometer.

**Results:**

In the study sample there were 91 (45.5%) males and 109 (54.5%) females with overall mean age of 34 years (±11.69). Predominant respiratory symptom was phlegm (19%) followed by cough (17.5%), wheeze (14%) and dyspnea (10.5%). Prevalence of physician diagnosed and self-reported asthma was 5.5% and 9.5% respectively. Frequency of obstructive pattern on spirometry was 28.72% and that of restrictive pattern was 19.68%. After adjustment for age, gender, socioeconomic status, spoken dialect, education, smoking status, height, weight and arsenic in drinking water, FVC was significantly reduced for phlegm (OR 3.01; 95% CI: 1.14 – 7.94), wheeze (OR 7.22; 95% CI: 2.52 – 20.67) and shortness of breath (OR 4.91; 95% CI: 1.57 – 15.36); and FEV_1_ was significantly reduced for cough (OR 2.69; 95% CI: 1.12 – 6.43), phlegm (OR 3.01; 95% CI: 1.26 – 7.16) and wheeze (OR 10.77; 95% CI: 3.45 – 33.6). Presence of respiratory symptoms was significantly associated with restrictive and/or obstructive patterns after controlling for confounders. Similar findings were observed through linear regression where respiratory symptoms were found to be significantly associated with decrements in lung volumes. Specificity and positive predictive values were found to be higher for all the symptoms compared to sensitivity and negative predictive values.

**Conclusion:**

Symptoms based respiratory questionnaires are a valuable tool for screening of respiratory symptoms in resource poor, rural community setting.

## Background

Spirometry is recommended as basis for diagnosing impaired lung function [1,2] but lack of spirometry equipment and expertise to use it in primary care settings of developing countries make it an unfeasible option [3]. In these settings, respiratory symptom-based questionnaires can be a simple and cost effective tool enabling identification and diagnosis of patients with respiratory illnesses. A number of respiratory questionnaires containing questions about symptoms of chronic obstructive pulmonary disease (COPD) and asthma have been developed [4-8]. American Thoracic Society Division of Lung Disease questionnaire (ATS – DLD-78A) [9] is a commonly used questionnaire for identifying the respiratory symptoms. It contains questions regarding frequent and chronic respiratory symptoms including cough, phlegm, wheeze and shortness of breath. It has been used after translation into local languages in a number of studies [10-13]. When used in conjunction with spirometry, symptoms based questionnaires can be a useful adjunct in the screening of population for respiratory illnesses [14].

COPD and asthma are chronic debilitating illnesses that profoundly affect the quality of daily life. Globally asthma is responsible for 1.8 million YLD (years lived with disability) whereas COPD is the 4^th^ leading cause of death [15]. COPD is characterized by partial air flow limitation which is not fully reversible. If left untreated, COPD can become a progressive and disabling disease [1]. Under-diagnosis of these symptoms is a problem worldwide [1] and lack of required training, failure to suspect symptoms by physicians and late presentation of patients to physician are the main causes for this under-diagnosis [14,16]. In developing countries including Pakistan, issues of accessibility and scarcity of resources in primary care setting necessitate the use of alternative measures particularly in distant rural areas. We used a modified version of the American thoracic society division of lung diseases questionnaire (ATS-DLD-78A) to identify the presence of respiratory symptoms. The objective of this study was to determine the correlation of respiratory symptoms and spirometric lung patterns as well as to determine the validity of the ATS respiratory questionnaire in a rural community setting of Sindh, Pakistan.

## Methods

### Study design and setting

This cross sectional study was conducted in two villages (Mehtani and Mian Jan Muhammad Abbasi) of union Council Agra, taluka (sub-district) Gambat, district Khairpur, Sindh between January – March 2009. The detailed methodology is given elsewhere [12] however; briefly, there were 200 adult (≥ 18 years) men and women who participated in this study. The population of union council Agra is approximately 21, 749 [12], the majority speaks Sindhi while Saraiki is a commonly spoken dialect which is itself derived from Sindhi, having 85% lexical similarity with Sindhi. Farming is the main occupation in this area [17].

These villages are located close to the banks of River Indus where there is a high proportion of drinking water sources contaminated with arsenic [18].

### Respiratory questionnaire

We used a modified version of the American thoracic society division of lung disease questionnaire (ATS-DLD 1978) to record the presence of respiratory symptoms. It included questions regarding frequent cough (defined as presence of cough on most days for 3 consecutive months or more during the year), chronic cough (defined as presence of cough for 3 consecutive months in 2 consecutive years), frequent phlegm (defined as bringing up phlegm on most days of month, for 3 consecutive months or more in a year), chronic phlegm (presence of phlegm for 3 consecutive months in 2 consecutive years), frequent wheezing (whistling sound heard on expiration within 2 years), chronic wheezing (whistling sounds heard on expiration more than 2 years), shortness of breath Grade I (shortness of breath, when hurrying on the level or walking up a slight hill) and Grade II (dyspnea defined as: walk slower than people of the same age on the level because of breathlessness or has to stop for breathing when walking at own pace on level), self-reported asthma (defined as respondent ever had asthma) and physician diagnosed asthma (defined as asthma confirmed by a doctor). Questions pertaining to socio-economic status (monthly household income and land ownership), education, occupation, type of kitchen, type of fuel used for cooking and smoking cigarettes and *beerie* (small locally made cigarette without filter commonly smoked in South Asia) or *huqqa* (local name for water-pipe) were added to the questionnaire.

The questionnaire was translated into Sindhi and back translated into English; it was then pretested in another Sindhi speaking community before use in this study. During the data collection the study team was particularly trained to make sure that the respondents were able to truly comprehend the meaning of all questions, especially those pertaining to presence of respiratory symptoms, such as wheeze.

### Lung function measurement

A portable Spirometer (Vitalograph New Alpha 6000; Vitalograph Ltd., Buckingham, England) was used for performing lung function measurements in accordance with the American Thoracic Society guidelines [19]. The age, height and sex predicted values of Forced Vital Capacity (FVC), forced expiratory volume in one second (FEV_1_) and their ratio (FEV_1_/FVC) were recorded in milliliters (ml) and percentages. European Respiratory Society (ERS) equations were used which were adjusted for Asian population using the correction factor of 0.9 [20]. Predicted percentage of ≥ 80% for FVC and FEV_1_ and FEV_1_/FVC ratio of ≥0.7 were considered as cut off for lung function tests to be normal. These cut-offs are generally used internationally for categorizing lung volumes as normal or abnormal [21]. Obstructive lung function was defined as having FEV_1_ <80% and FEV_1_/FVC <70% and restrictive lung function was defined as having FEV_1_ <80% and FEV_1_/FVC >70% [2].

Participants were explained the procedure of spirometry in detail and were allowed to practice until they felt comfortable. Participants were asked to refrain from smoking for at least 1 hour prior to the procedure. Spirometry was conducted in standing position without nose clips and ATS repeatability criteria were used for quality assessment of the spirometry maneuver. Results of three acceptable readings were recorded and best of the three readings was used for analysis in this study. Spirometry was conducted by a trained technician who was also well versed with local language. Spirometry reports were further assessed by a pulmonologist for quality assurance.

### Ethical considerations

Study approval was taken from ethics review committee of Aga Khan University, Karachi. Verbal and written informed consent was taken from the participants.

### Statistical analysis

Data was entered into Epidata 3.1 and analyzed using SPSS 19 for windows. Descriptive statistics were calculated for socio-demographic variables, respiratory symptoms and lung volumes.

Percentage predicted lung volumes were entered as continuous variables and independent samples t-test was applied to determine significant differences in lung function decrements according to presence and absence of respiratory symptoms.

Logistic regression analysis was performed to determine crude and adjusted odds ratio of respiratory symptoms with percentage predicted lung volumes entered as dichotomous variables (FVC > 80% *vs* < 80%; FEV_1_ > 80% *vs* < 80% & FEV_1_/FVC ratio > 0.7 *vs* < 0.7). In multivariate model, adjustment was done for age, height, weight, sex, smoking status, spoken dialect, education, socio-economic status (SES) and arsenic levels in drinking water.

Furthermore linear regression analysis was also performed separately for FEV1, FVC and FEV1/FVC ratio in order to determine the association of percentage predicted lung volumes with respiratory symptoms. Validity of the ATS respiratory questionnaire along with predictive values were also calculated for obstructive and restrictive lung patterns separately.

## Results

Frequencies of socio-demographic, anthropometric characteristics and lung function of study participants are given in Table 1. Majority of the participants were uneducated (59.7%) and predominant type of occupation was farmers and laborers (28.5%) Approximately 62.5% were owners of agricultural land and median monthly household income was 8000 Pakistani rupees (US$ 98). Mean height was 160.47 cm (±8.73) and mean weight was 59.29 kg (±12.93). Approximately 21.5% were smokers, predominant type of fuel used for cooking was biomass i.e. firewood and cow dung (78%), while the commonly prevalent type of kitchen was open air and sheltered (roofs commonly made of wood/straws) (82%).

**Table 1:** Socio-demographic, anthropometric characteristics and lung function of the study population

	***Male n (%)***	***Female n (%)***	***Total n (%)***
***Variable***			
Study population	91 (45.5)	109 (54.5)	200 (100)
Education			
Uneducated	22	97	119 (59.7)
Educated	68	12	80 (42.3)
Occupation			
House wife	0	108	108 (54)
Farmers/laborers	57	0	57 (28.5)
Govt./private service	16	0	16 (8)
Retired/unemployed	10	0	10 (5)
Smoking status ^1^			
Never smoker	61	103	164 (82)
Ever smoker	30	6	36 (18)
Dialect			
Sindhi	30	34	64 (32)
Saraiki	61	75	136 (68)
Agricultural land ownership ^2^			
Do not own land	35	40	75 (37.5)
Own land	56	69	125(62.5)
			
Monthly household income(PKR) ^3^***;Mean (SD)***	10156 (±6600)	7610 (±6519)	8000 (±5000)
Height (cm) ***;Mean (SD)***	167.51 (±6.02)	154.75 (±5.81)	160.47 (±8.63)
Weight (kg) ***;Mean (SD)***	64.73 (±13.53)	55.02 (±10.61)	59.29 (±12.93)
Age (years) ***;Mean (SD)***	33.12 (±13.23)	34.48 (±10.22)	34 (±11.69)
Duration living in house (years) ***;Mean (SD)***	28.76 (±13.93)	32.23 (±13.71)	31 (±13.83)
**%** Predicted Spirometric lung volumes in milliliters (N 188) ^4^			
FVC ^5^***;Mean (SD)***	95.90 (±19.84)	97.11 (±21.94)	96.55 (±20.95)
FEV_1_^6^***;Mean (SD)***	82.61 (±20.96)	82.02 (±21.62)	82.29 (±21.26)
FEV_1_**/** FVC ***;Mean (SD)***	88.92 (±11.42)	88.50 (±12.52)	88.69 (±12)

^1.^ Smoking status defined as ever/never smoker of cigarette, beerie or huqqa.

^2.^ Land unit defined as Jaraib where 1 acre = 2 Jaraib.

^3.^ Income reported in Pakistani rupees (PKR), 1 US $ = 90 PKR, median and interquartile range are given due to skewed distribution.

^4.^ Sample size reduced due to missing or incomplete information.

^5.^ Forced vital capacity.

^6.^ Forced expiratory volume in 1 second.

On the basis of quality assessment of spirometry reports 12 entries were excluded from the analysis of lung volumes. Mean of % predicted lungs volumes for 188 participants were recorded as: FVC 96.55 (±20.95), FEV_1_ 82.29 (±21.26), FEV_1_/FVC ratio 88.69 (±12).

Predominant type of respiratory complaint was Phlegm (19%) followed by cough (17.5%), wheeze (14%) and shortness of breath (10.5%). Frequency of physician diagnosed asthma was 5.5% (Table 2). Frequency of Obstructive pattern on spirometry was 28.57.5% and that of restrictive pattern was 19.57%.

**Table 2:** Frequency distribution of respiratory symptoms, asthma and lung function among study population in taluka Gambat district Khairpur, Sindh, Pakistan

***Prevalence of respiratory symptoms (Total sample N 200)***	***n***	***(%)***
Cough ^7^	35	17.5
Phlegm ^8^	38	19
Wheeze	28	14
Shortness of breath ^9^	21	10.5
Self-reported asthma ^10^	19	9.5
Physician diagnosed asthma ^11^	11	5.5
***Frequency of Lung function patterns (Total sample N 188)***		
Normal	98	52.12
Obstructive ^12^	54	28.72
Restrictive ^13^	37	19.68

^7.^ Chronic cough defined as cough on most days of month, for 3 consecutive months or more in a year.

^8.^ Chronic phlegm defined as presence of phlegm for 3 consecutive months in 2 consecutive years.

^9.^ Participants were asked: do you have to walk slower than people of your age on the level because of breathlessness?

^10.^ Participants were asked if they ever had asthma.

^11.^ Physician diagnosed asthma defined as asthma confirmed by a doctor.

^12.^ Defined as having FEV_1_ <80% and FEV_1_/FVC <70%.

^13.^ Defined as having FEV_1_ <80% and FEV_1_/FVC >70%.

**Figure 1:**
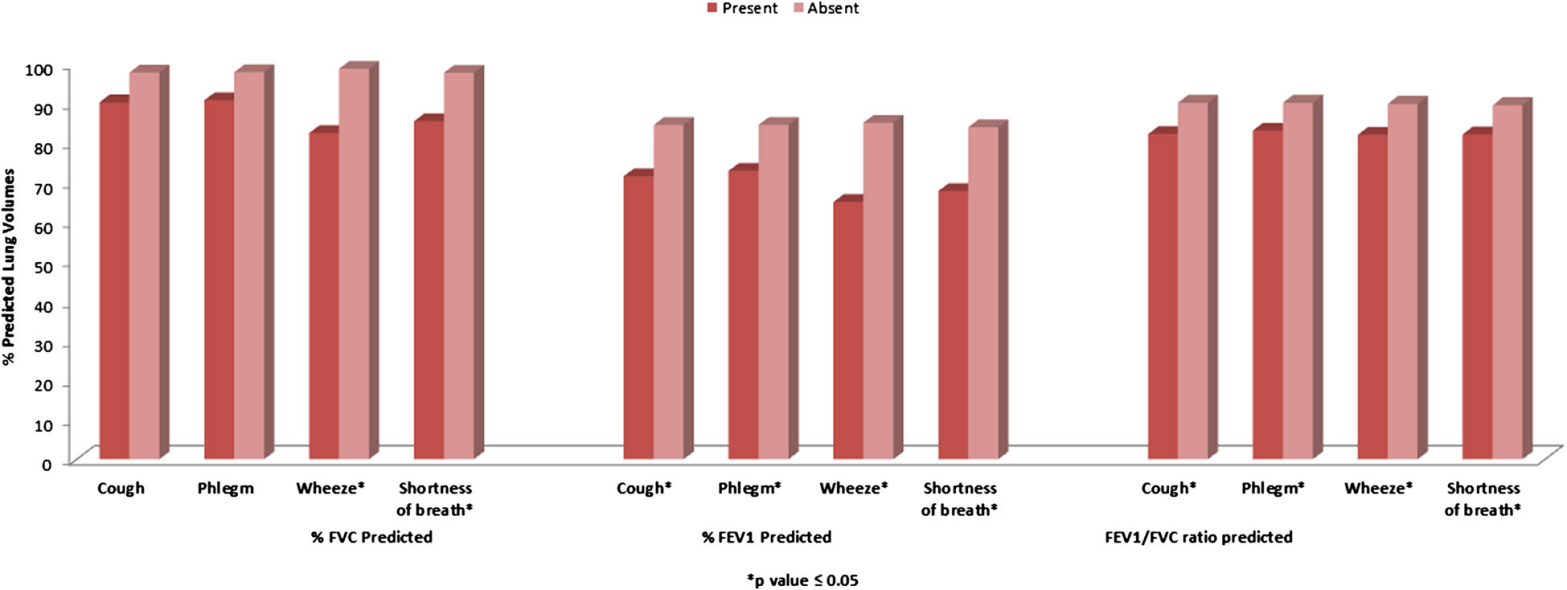
Difference in lung function of the study population according to presence and absence of respiratory symptoms.

A trend of reduced lung function (predicted FVC, FEV_1_ and FEV_1_/FVC ratio) was observed among participants with respiratory symptoms compared to those without symptoms (Figure 1).

In univariate logistic regression, decrements in percentage predicted FVC were significant for all symptoms except cough.

Percentage predicted FEV_1_ was significantly reduced for all symptoms except shortness of breath (Crude OR 4.8, 95% CI: 1.32 – 17.45). Statistically significant reductions in FEV_1_/FVC ratio were found for cough (Crude OR 3.47, 95% CI: 1.05 – 11.41) and shortness of breath (Crude OR 4.8, 95% CI: 1.32 – 17.45) (Table 3).

**Table 3:** Crude and adjusted logistic regression analysis of respiratory symptoms with spirometric lung function patterns, taluka Gambat district Khairpur, Sindh, Pakistan

	**Crude OR**	Adjusted OR*	**P - value**
	**(Confidence interval)**	**(Confidence interval)**	
**% Predicted FVC volume**^**14**^			
Cough ^15^	2.02 (0.86 – 4.72)	2.33 (0.87 – 6.19)	0.089
Phlegm ^16^	2.35 (1.04 – 5.27)	3.01 (1.14 – 7.94)	<0.026
Wheeze	4.34 (1.81 – 10.42)	7.22 (2.52 – 20.67)	<0.001
Shortness of breath ^17^	3.28 (1.22 – 8.84)	4.91(1.57 – 15.36)	<0.006
**% Predicted FEV**_**1**_** volumes**^**18**^			
Cough	2.44 (1.11 – 5.35)	2.69 (1.12 – 6.43)	0.026
Phlegm	2.33 (1.1 – 4.9)	3.01 (1.26 – 7.16)	0.013
Wheeze	6.73 (2.41 – 18.76)	10.77 (3.45 – 33.6)	<0.001
Shortness of breath	2.35 (0.88 – 6.28)	2.23 (0.78– 6.36)	0.131
**% Predicted FEV**_**1**_**/FVC ratio**^**19**^			
Cough	3.47 (1.05 – 11.41)	3.02 (0.62 – 14.65)	0.169
Phlegm	2.94 (0.90 – 9.60)	3.85 (0.68 – 21.8)	0.127
Wheeze	3.13 (0.88 – 11.03)	4.28 (0.73 – 24.81)	0.105
Shortness of breath	4.8 (1.32 – 17.45)	3.45 (0.69 – 17.15)	0.130
**Presence of obstructive**^**20**^**and/or restrictive lung disease (Total sample N 188)**			
Cough	3.30 (1.43 – 7.06)	3.24 (1.18 – 8.79)	0.021
Phlegm	3.48 (1.56 – 7.72)	5.33 (1.94 – 14.69)	0.001
Wheeze	7.41(2.44 – 22.49)	12.10 (3.34 – 43.79)	<0.001
Shortness of breath	3.34 (1.15 – 9.70)	3.02 (0.93 – 9.87)	0.67

*Adjusted for age, height, weight, sex, smoking status, education, socioeconomic status, spoken dialect and arsenic levels.

^14.^ Entered as dichotomous variable >80% and <80%.

^15.^ Chronic cough defined as cough on most days of month, for 3 consecutive months or more in a year.

^16.^ Chronic phlegm defined as presence of phlegm for 3 consecutive months in 2 consecutive years.

^17.^ Participants were asked: do you have to walk slower than people of your age on the level because of breathlessness?

^18.^ Entered as dichotomous variable >80% and <80%.

^19.^ Entered as dichotomous variable >70% and <70%.

^20.^ Obstructive lung disease: defined as having FEV_1_ <80% and FEV_1_/FVC <70%; restrictive lung disease: defined as having FEV_1_ <80% and FEV_1_/FVC >70%, sample size reduced due to missing/incomplete information; a composite variable containing both obstructive and restrictive lung function was created.

After adjustment for confounders in multivariate analysis, FVC was significantly reduced for phlegm (OR 3.01; 95% CI: 1.14 – 7.94), wheeze (OR 7.22; 95% CI: 2.52 – 20.67) and shortness of breath (OR 4.91; 95% CI: 1.57 – 15.36); FEV_1_ was significantly reduced for cough (OR 2.69; 95% CI: 1.12 – 6.43), phlegm (OR 3.01; 95% CI: 1.26 – 7.16) and wheeze (OR 10.77; 95% CI: 3.45 – 33.6). No significant reduction was found in FEV_1_/FVC ratio for any symptom (Table 3). After adjustment, risk of obstructive and/or restrictive lung disease was significant for all symptoms except shortness of breath (Adjusted OR 3.02, 95% CI: 0.93 – 9.87) (Table 3). On linear regression significant association was found for decrements in lung function for all respiratory symptoms, except wheeze (Table 4).

**Table 4:** Crude and adjusted linear regression analysis of respiratory symptoms with percentage predicted lung volumes, taluka Gambat district Khairpur, Sindh, Pakistan

	**Crude beta coefficient (Confidence interval)**	Adjusted beta coefficient* (Confidence interval)	**P - value**
**% Predicted FVC volumes**			
Cough ^21^	−7.537 (−15.53 – 0.45)	−9.353(−17.30 – -1.39)	0.021
Phlegm ^22^	−7.100 (−14.73 – 0.53)	−9.792 (−17.55 – -2.02)	0.014
Wheeze	−16.190 (−24.65 – -7.72)	−17.876 (−26.13 – -9.61)	< 0.001
Shortness of breath ^23^	−12.191 (−22.09 – -2.28)	−8.965 (−19.09 – -1.16)	0.082
**% Predicted FEV**_**1**_** volumes**			
Cough	−12.969 (−20.91 – -5.02)	−14.510 (−22.52 – -6.49)	<0.001
Phlegm	−11.631 (−19.24 – -4.01)	−13.814 (−21.70 – -5.92)	0.001
Wheeze	−20.068 (−28.46 – -11.66)	−22.120 (−30.41 – -13.82)	< 0.001
Shortness of breath	−13.967 (−24.59 – -3.33)	−15.176 (−25.08 – -5.267)	0.003
**% Predicted FEV**_**1**_**/FVC ratio**			
Cough	−7.876 (−12.34 – -3.41)	−6.841 (−11.16 – -2.51)	0.002
Phlegm	−6.936 (−11.22 – -2.65)	−6.778 (−11.09 – -2.46)	0.002
Wheeze	−7.765 (−12.65 – -2.87)	−8.245 (−13.21 – -3.27)	0.001
Shortness of breath	−7.327 (−12.97 – -1.68)	−7.403 (−13.21 – -1.59)	0.013

*Adjusted for age, height, weight, sex, smoking status, education, socioeconomic status, spoken dialect and arsenic levels.

^21.^ Chronic cough defined as cough on most days of month, for 3 consecutive months or more in a year.

^22.^ Chronic phlegm defined as presence of phlegm for 3 consecutive months in 2 consecutive years.

^23.^ Participants were asked: do you have to walk slower than people of your age on the level because of breathlessness (SOB grade II).

The negative predictive values and specificity of all the symptoms were generally higher with respect to obstructive and restrictive lung patterns compared to positive predictive values and sensitivity respectively (Table 5 and Table 6).

**Table 5:** Sensitivity, specificity and predictive values of respiratory symptoms and asthma in relation to obstructive spirometric interpretation, taluka Gambat district Khairpur, Sindh, Pakistan

**Symptoms**	**Positive predictive value (%)**	**Negative predictive value (%)**	**Sensitivity (%)**	**Specificity (%)**
Frequent cough ^24^	40.62	73.88	24.07	85.92
Chronic cough ^25^	53.33	73.56	14.8	94.81
Frequent phlegm ^26^	36.11	73.20	24.07	82.96
Chronic phlegm ^27^	42.10	72.94	14.80	91.85
Frequent wheeze ^28^	34.61	72.39	16.66	87.40
Chronic wheeze ^29^	36.48	72.35	12.96	91.11
Shortness of breath grade I ^30^	32.35	72.25	20.37	82.96
Shortness of breath grade II ^31^	30.00	71.59	11.11	89.62
Composite variable for frequent symptoms ^32^	28.33	71.31	31.48	68.14
Composite variable for chronic symptoms ^33^	32.43	72.36	22.22	81.48
Self-reported asthma ^34^	36.84	72.35	12.96	91.11
Physician diagnosed asthma ^35^	27.27	71.34	5.55	94.07

^24.^ Frequent cough defined as presence of cough on most days for 3 consecutive months or more during the year.

^25.^ Chronic cough defined as presence of cough for 3 consecutive months in 2 consecutive years.

^26.^ Frequent phlegm defined as bringing up phlegm on most days of month, for 3 consecutive months or more in a year.

^27.^ Chronic phlegm defined as presence of phlegm for 3 consecutive months in 2 consecutive years.

^28.^ Frequent wheezing defined as whistling sound heard on expiration within 2 years.

^29.^ Chronic wheezing defined as whistling sounds heard on expiration more than 2 years.

^30.^ Shortness of breath Grade I defined as shortness of breath, when hurrying on the level or walking up a slight hill.

^31.^ Shortness of breath Grade II defined as: walk slower than people of the same age on the level because of breathlessness or has to stop for breathing when walking at own pace on level.

^32.^ Composite variable for frequent symptoms: Frequent (cough + phlegm + wheeze + shortness of breath).

^33.^ Composite variable for chronic symptoms: Chronic (cough + phlegm + wheeze + shortness of breath).

^34.^ Self-reported asthma defined as respondent ever had asthma.

^35.^ Physician diagnosed asthma defined as asthma confirmed by a doctor.

**Table 6:** Sensitivity, specificity and predictive values of respiratory symptoms and asthma in relation to restrictive spirometric interpretation, taluka Gambat district Khairpur, Sindh, Pakistan

**Symptoms**	**Positive predictive value (%)**	**Negative predictive value (%)**	**Sensitivity (%)**	**Specificity (%)**
Frequent cough ^36^	31.25	82.80	27.02	85.52
Chronic cough ^37^	40.00	82.18	16.21	94
Frequent phlegm ^38^	36.11	84.31	35.13	84.86
Chronic phlegm ^39^	42.10	82.94	21.62	92.76
Frequent wheeze ^40^	50.00	85.27	35.13	91.44
Chronic wheeze ^41^	47.36	83.52	18.91	93.42
Shortness of breath grade I ^42^	35.29	83.87	32.43	85.52
Shortness of breath grade II ^43^	40.00	82.84	21.62	92.10
Composite variable for frequent symptoms ^44^	31.66	86.04	51.35	73.02
Composite variable for chronic symptoms ^45^	37.83	84.86	37.83	84.86
Self-reported asthma ^46^	31.57	81.76	16.21	91.44
Physician diagnosed asthma ^47^	45.45	82.02	13.51	96.05

^36.^ Frequent cough defined as presence of cough on most days for 3 consecutive months or more during the year.

^37.^ Chronic cough defined as presence of cough for 3 consecutive months in 2 consecutive years.

^38.^ Frequent phlegm defined as bringing up phlegm on most days of month, for 3 consecutive months or more in a year.

^39.^ Chronic phlegm defined as presence of phlegm for 3 consecutive months in 2 consecutive years.

^40.^ Frequent wheezing defined as whistling sound heard on expiration within 2 years.

^41.^ Chronic wheezing defined as whistling sounds heard on expiration more than 2 years.

^42.^ Shortness of breath Grade I defined as shortness of breath, when hurrying on the level or walking up a slight hill.

^43.^ Shortness of breath Grade II defined as: walk slower than people of the same age on the level because of breathlessness or has to stop for breathing when walking at own pace on level.

^44.^ Composite variable for frequent symptoms: Frequent (cough + phlegm + wheeze + shortness of breath).

^45.^ Composite variable for chronic symptoms: Chronic (cough + phlegm + wheeze + shortness of breath).

^46.^ Self-reported asthma defined as respondent ever had asthma.

^47.^ Physician diagnosed asthma defined as asthma confirmed by a doctor.

## Discussion

We believe that this is amongst the first studies to report validation of ATS respiratory questionnaire in a local language (Sindhi) in rural setting of Pakistan. The study shows that respiratory symptoms of cough, phlegm, wheeze and shortness of breath are significantly correlated with reduced lung function and this trend was found consistently for all lung volumes (FVC, FEV_1_ and FEV_1_/FVC). This correlation suggests that the presence of respiratory symptoms is an important predictor of impaired lung function and use of standardized respiratory questionnaires like ATS questionnaire therefore is an effective tool in estimating the burden of respiratory symptoms in a rural community setting. This finding has important Public Health implications for resource limited settings because use of such standardized questionnaires can serve as a cost saving tool in the diagnosis of respiratory symptoms without need of additional resources.

This study has reported significant association of all symptoms with both obstructive and restrictive lung patterns. These findings are similar to those reported in other studies. A number of studies conducted on different occupational groups have shown association of selective symptoms with reduced lung function. In one study symptoms of cough and phlegm were found to be significantly associated with obstructive pattern as opposed to wheeze or phlegm [22]. While the predominant associations of selective symptoms with lung function changes reported in these studies can be attributed to specific occupational exposures, symptoms identified by questionnaires successfully predicted the impaired lung function which was verified by spirometry.

We collected the data on major confounders and adjusted the results in final analysis. The study area from where the sample was selected has high arsenic concentration in ground water. Studies have reported important role of arsenic in reduced lung function [12]. We measured the levels of arsenic in drinking water sources which were adjusted in final results. Adjustment was also done for smoking, type of fuel used for cooking and type of kitchen which are independent factors of respiratory illnesses.

In this study, we also report the prevalence of physician diagnosed and self-reported asthma as 5.5% and 9.5% respectively. There is paucity of representative data regarding prevalence of asthma in Pakistan. Few studies that were conducted have estimated the burden of asthma among selective groups of population including children [23,24] and some occupational groups [25] whereas our study estimated the burden of asthma in general population of adults. Prevalence reported in this study is somewhat similar to that reported using similar definition by WHO for Pakistan and neighboring countries like India, Bangladesh and Srilanka where it ranged between 2.6% to 3.16% [26]. Possible reasons for variation in asthma prevalence in different studies could include the variation in operational definition of asthma amongst different studies. Use of definition such as physician diagnosed asthma may be appropriate in developed countries; however, asthma prevalence estimated on the basis of this definition can vary greatly in developing countries depending on availability of and access to health care facilities and required medicines [26]. Random sampling error may be an alternate explanation for variation in prevalence in this study compared to the other studies. Our study population was exposed to arsenic through underground drinking water and prolonged exposure to arsenic is being increasingly recognized to affect the lung function [12,27] however, further research is warranted to explore such associations. Although our study reports the prevalence of asthma from a localized community setting of a rural area, we believe that these estimates may be generalizable to similar rural settings in Pakistan especially along the river Indus since similar risk factors for respiratory illnesses are prevalent in these areas.

In addition to arsenic exposure, several other factors could be related to the presence of respiratory symptoms and asthma among this study population especially the use of biomass fuel for cooking and the type of kitchen being used. However, this study was not powered to detect the risk factors associated with respiratory symptoms due to limited sample size.

This study has few limitations that need to be considered. Due to cross sectional nature of the study, it is difficult to establish causal association between impaired lung function and the respiratory symptoms. Nevertheless, the consistent trend of decrement in lung volumes is of importance and provides basis for further research. This was a pilot study with a small sample size however, as a preliminary research, this study significantly adds to the scarcely available evidence regarding ATS respiratory questionnaire validation in rural community settings especially in the developing countries. We used spirometry as an objective measure of lung function to compare with the respiratory questionnaire. Spirometry has an added advantage of identifying impaired lung function among those who do not have manifest symptoms and it may not be possible to capture such impairment by use of questionnaire alone, as indicated by the results of our study, that show low sensitivity and positive predictive values while high specificity and negative predictive values for all the frequent or less severe as well as chronic or more severe symptoms in the ATS questionnaire. This finding is consistent with studies using different questionnaires that have also reported low sensitivity and high specificity [28].

There is scarce literature regarding comparison of ATS questionnaire with other respiratory questionnaires although available studies have reported analogous results. Asthma related items of ATS questionnaire have been compared with those of Medical research council questionnaire (MRC) and National Heart and Lung Institute questionnaire (NHLI) yielding similar responses [28]. Therefore we believe that ATS questionnaire is a valid tool for assessment of respiratory symptoms in rural community settings.

## Conclusion

Respiratory symptoms of cough, wheeze, dyspnea and phlegm are significantly correlated with reduced lung function and should be strongly emphasized in clinical history for assessment of respiratory health. These symptoms are important predictor of obstructive and restrictive lung function independent of risk of smoking. Validated respiratory questionnaires like ATS questionnaire are an effective tool in the diagnosis of respiratory symptoms and can be a useful adjunct to spirometry in community setting of resource poor countries for screening of respiratory illness.

## Abbreviations

MRC: Medical research council; NHLI: National Heart and Lung Institute; ATS-DLD: American thoracic society – division of lung diseases; WHO: World health organization; FVC: Forced vital capacity; FEV: Forced expiratory volume; SES: Socio-economic status; COPD: Chronic obstructive pulmonary disease; YLD: Years lived with disability.

## Competing interests

All authors declare that they have no competing interests.

## Authors' contributions

INA and AAN conceived the study; INA and AA performed the study measure and AAN supervised the process. IN, AAN and AA performed the data analysis. INA and AAN wrote the first draft of the paper to which all authors subsequently made contributions. All authors read and approved the final manuscript.

## Pre-publication history

The pre-publication history for this paper can be accessed here:

http://www.biomedcentral.com/1471-2466/12/81/prepub
